# Cocaine vaccine dAd5GNE protects against moderate daily and high-dose “binge” cocaine use

**DOI:** 10.1371/journal.pone.0239780

**Published:** 2020-11-30

**Authors:** David F. Havlicek, Jonathan B. Rosenberg, Bishnu P. De, Martin J. Hicks, Dolan Sondhi, Stephen M. Kaminsky, Ronald G. Crystal

**Affiliations:** Department of Genetic Medicine, Weill Cornell Medical College, New York, New York, United States of America; CNRS, FRANCE

## Abstract

The cocaine vaccine dAd5GNE is comprised of a disrupted serotype 5 adenovirus gene therapy vector covalently conjugated to the cocaine analog GNE. The vaccine evokes a high titer of circulating anti-cocaine antibodies that prevent cocaine from reaching its cognate receptors in the central nervous system. Prior studies have demonstrated the efficacy of dAd5GNE in models of occasional, moderate cocaine use. However, previous studies have not sufficiently evaluated the efficacy of dAd5GNE in models of the repetitive and high-dose “binge” use patterns common in human addicts. In the present study, we evaluated the capacity of dAd5GNE vaccination to protect against “binge” cocaine use and circumstances where vaccinated addicts attempt to override the vaccine. We modeled repetitive daily cocaine use in vaccinated Balb/c mice and African green monkeys, and evaluated high-dose “binge” scenarios in Balb/c mice. In each model of daily use the dAd5GNE vaccine prevented cocaine from reaching the central nervous system. In the high-dose “binge” model, vaccination decreased cocaine-induced hyperactivity and reduced the number of cocaine-induced seizures. Based on this data and our prior data in rodents and nonhuman primates, we have initiated a clinical trial evaluating the dAd5GNE anti-cocaine vaccine as a potential therapy for cocaine addicts who wish to stop cocaine use. If dAd5GNE vaccination is safe and produces high anti-cocaine antibody titers in the clinic, we hypothesize that the vaccine will restrict the access of cocaine to the central nervous system and inhibit cocaine-induced “highs” even in the context of moderate daily and high-dose “binge” use that might otherwise cause a drug-induced overdose.

## Introduction

In the United States, over one million people use cocaine regularly [1] and cocaine contributes to >500,000 emergency room visits per yr [2]. Cocaine use is a serious public health concern and there are currently no FDA approved therapies to treat cocaine addiction. A vaccine that elicits high titers of anti-cocaine antibodies capable of binding to cocaine would prevent cocaine from passing across the blood-brain barrier, thus reducing the reinforcing effects of a perceived high and serving as a potential clinical therapeutic for individuals trying to overcome addiction [3–6].

The cocaine vaccine dAd5GNE is comprised of the cocaine analog GNE covalently linked to the proteins of a disrupted E1^-^ E3^-^ serotype 5 adenovirus (Ad5) [7]. Adenovirus has a favorable safety profile demonstrated in clinical studies and stimulates a robust immune response which is cross-targeted to the cocaine analog GNE when covalently linked [8–12]. The adenovirus capsid is disrupted to expose additional sites for GNE coupling and was more potent immunologically [13]. The vaccine dAd5GNE produces high titers of anti-cocaine antibodies, which block cocaine from reaching the central nervous system (CNS) of mice, rats, and nonhuman primates (NHPs) [7, 10–13]. In these models, vaccination reduced cocaine-induced hyperactivity and successfully curtailed cocaine self-administration in addicted animals [7, 13]. Furthermore, dAd5GNE immunization prevented cocaine access to peripheral organs not restricted by the blood-brain barrier [10] and was effective even in the context of pre-existing Ad5 immunity, which is prevalent in the human population [12].

The goal of an anti-cocaine vaccine is to block the ability of cocaine to reach the brain, thus obviating the cocaine “high” and the reinforcing effects that promote addiction [3]. Previous studies have shown that dAd5GNE effectively meets this challenge [7, 10–13]. However, given that cocaine users often use the drug in a “binge” pattern, taking the drug repeatedly, at increasingly higher doses, within a relatively short period of time [14], an efficacious vaccine would need to have the capacity to block multiple cocaine doses [3]. Previous studies have shown that dAd5GNE vaccination reduces cocaine levels in the brain [10, 11] and reduces cocaine-induced hyperactivity [7] following multiple drug exposures. However, models of daily cocaine use have not been sufficiently evaluated. Given that a high percentage of cocaine addicts use the drug daily or more frequently [15–18], successful clinical translation will require the vaccine to be effective against repetitive cocaine-dosing regimens.

Another challenge for a vaccine to treat cocaine addiction is the amount of the drug dose. While moderate 0.1 mg/kg intravenous doses of cocaine produce a cocaine-induced “high” in clinical settings [19], many cocaine addicts use higher doses to maintain or enhance their “high” [17]. While previous studies have demonstrated vaccine efficacy at moderate cocaine doses (0.0625 mg/kg– 2 mg/kg intravenous (iv) or 15 mg/kg intraperitoneal) [7, 10–13] vaccine efficacy has not been assessed in a high-dose intravenous administration model (> 2mg/kg). Evaluation of vaccine efficacy at high doses is of particular importance for clinical translation, as vaccinees may increase the cocaine dose needed to overcome the vaccine [20, 21]. Therefore, the capacity to block very high doses of cocaine is a critical performance characteristic for dAd5GNE as a clinical therapeutic.

Cocaine users may inhale the drug or administer it by oral, intranasal, or intravenous routes of administration. For these studies we evaluated the intravenous route of administration because it has a fast onset and the highest bioavailability of all routes [22]. Based on these characteristics intravenous administration likely provides the greatest challenge to vaccine efficacy. Additionally, these studies require accurate cocaine dosing for evaluation of vaccine efficacy. Intravenous administration likely provides the most accurate route of administration for animal studies. In the current study we modeled low to moderate repeated daily intravenous cocaine use in mice and nonhuman primates and modeled high-dose intravenous “binge” use in mice. While these intravenous administrations likely provide the greatest challenge to vaccine efficacy, we expect that the vaccine will remain effective for other routes of cocaine administration.

## Methods

### Vaccine production

The base of the dAd5GNE vaccine, Ad5LacZ, is a recombinant E1a‾, partial E1b‾, and E3‾ serotype 5 adenovirus gene therapy vector carrying the LacZ transgene, which was propagated and purified as described previously [23]. Inclusion of LacZ in the expression cassette was a strategic choice that allows for rapid and sensitive evaluation of viral infectivity before and after viral disruption [13]. The purified E1‾ E3‾ adenovirus was disrupted in 0.5% sodium dodecyl sulfate at 56°C for 45 sec and the cocaine analog GNE ([6-(2R,3S)-3-(benzoyloxy)-8-methyl-8-azabicyclo [3.2.1] octane-2-carboxoamido-hexanoic acid]) was activated overnight at 4°C with charging solution (1-ethyl-3-(3-dimethylaminopropyl) carbodiimide hydrochloride and sulfo-N-hydroxysulfosuccinimide in H_2_O and dimethylformamide). The charged GNE was conjugated to the capsid proteins of the disrupted Ad5 particles (300:1 GNE to Ad capsomere molar ratio) by overnight incubation at 4°C in phosphate-buffered saline (PBS, pH 7.4). The final conjugated product was dialyzed in sucrose/Tris buffer (10 mM Tris-HCl, 87 mM Sucrose, 21 mM MgCl_2_, 150 mM NaCl, in H_2_O, pH 7.8) prior to storage at −80°C. The final vaccine concentration was assayed by bicinchoninic acid assay (BCA) (Pierce Biotechnology, Rockford, IL). Disruption of the virus renders it non-infectious and exposes additional lysine residues to which the cocaine analog GNE can be conjugated. Successful viral disruption is confirmed utilizing a beta-galactosidase assay [13].

#### Animal studies

All animals were housed under pathogen-free conditions and studies were conducted under protocols reviewed and approved by the Weill Cornell Institutional Animal Care and Use Committee (# 2009–0017) following NIH guide for care and use of Laboratory animals. Monkeys were housed in paired-housed cages, fed twice daily, supplemented with fruit or vegetables daily, with access to water ad libitum, enriched with videos, toy and observed daily by the research specialists for general appearance, signs of toxicity, distress and changes in behavior.

#### Murine studies

Female BALB/c mice (Taconic, Germantown, NY) were immunized with 4 μg of dAd5GNE and 20% Adjuplex™ (Advanced BioAdjuvants, Omaha, NE) by 50 μl intramuscular injection to the quadriceps on wk 0, 4, and 8 (± 10 days). Naive mice were immunized with PBS and 20% Adjuplex™.

For assessment of anti-cocaine antibody titers, blood was collected from the transected tail vein at indicated time points. Blood was allowed to clot, centrifuged at 2500 *g* for 15 min and serum was stored at −20°C. Anti-cocaine antibody titers in serum were measured by ELISA as described previously [13]. Briefly, 96 well plates were coated with cocaine hapten conjugated to BSA. Plates were then washed and blocked before twofold serial dilutions of serum were added to each well. Plates were again washed, and anti-cocaine antibodies were detected with horseradish peroxidase-conjugated anti-IgG antibodies. Peroxidase substrate was added, and the absorbance was measured. Anti-cocaine antibody levels were calculated by interpolation of the plot of the log(OD) *vs* log(dilution), with a cutoff equal to twice the absorbance of background.

To assess cocaine biodistribution, mice were challenged with 0.1 mg/kg (~2.5 μg) cocaine in 100 μl of PBS delivered intravenously to the tail vein. The single cocaine challenge groups received cocaine on day 84 post vaccine prime. The daily cocaine challenge group received cocaine on days 82, 83, and 84. To ameliorate suffering mice were anesthetized by intraperitoneal injection of ketamine (100 mg/kg) and xylazine (10 mg/kg) 5 min prior to tail vein administration of 0.1 mg/kg (2.5 μg) cocaine containing 1.0 μCi ^3^H-cocaine (PerkinElmer, Waltham, MA). One min following cocaine administration, mice were sacrificed for brain collection. Brain tissue was homogenized in 1.5 ml of PBS and 300 μl of the brain homogenate was added to 5 ml of liquid scintillant (Ultima Gold, PerkinElmer), assayed for tritium, and normalized with a standard quenching curve, as described previously [13].

To evaluate cocaine-induced behavior, mice were challenged with 100 μl PBS delivered intravenously to the tail vein on wk 10. Cocaine challenge mice then received 1 mg/kg (25 μg; wk 11, 12, 13), 2 mg/kg (50 μg; wk 14, 16, 18), and 4 mg/kg (100 μg; wk 20) cocaine in 100 μl of PBS. PBS challenge mice received 100 μl PBS at each time point. The activity of mice was evaluated over 10 min directly following an intravenous PBS or cocaine injection. Mouse locomotor behavior was recorded using infrared beam-equipped, open-field, activity chambers (20 cm x 20 cm chamber; Accuscan Instruments, Columbus, OH). Mice were allowed to habituate to the room for 1 hr prior to each test. Locomotor activity was measured by Accuscan software as total ambulatory distance traveled in 10 min. Observers monitored the mice for epileptic activity throughout the challenge for decline in health (seizures), all mice in this, and all other studies here recovered. Following seizures, mice were removed from the activity chambers, placed in clean new home cages on clean towels and placed in a darker area of the room. After 30 min of return to normal activity, sternal recumbence and normal breathing, the mice were returned to their home cages with littermates and monitored 3X/day to assess recovery. Euthanasia was by Carbon dioxide overdose. Mice were exposed to 100% carbon dioxide at 5 PSI for >3 min, at a displacement rate of 30% chamber volume/minute, in mouse cage, in accordance with the American Veterinary Medical Association Guidelines.

Biodistribution studies were done at low to moderate cocaine doses for which differences in dopamine transporter occupancy have greater sensitivity to assessment [24]. Conversely, differences in the psychomotor effects of cocaine are detectable at relatively high doses whereas at the lower doses cocaine would not induce measurable hyper locomotor activity. Therefore the cocaine dose for each study was determined by the useful range of cocaine concentration sensitivity of the assay.

#### Nonhuman primate studies

African green monkeys were US sourced from the Wake Forest University Primate Center (Winston-Salem, NC), certified viral-free, housed in the Tri-institutional Animal Facility (Weill Cornell Medical College) and assigned to the following experimental groups: naive (n = 2 M/2 F) and dAd5GNE vaccinated (n = 6 M/6 F). Vaccinated animals were immunized with 50 to 200 μg of dAd5GNE and 20% Adjuplex™ by 500 μl intramuscular injection to the quadriceps on wk 0, 4, 8, 12, 16, and 20 (± 10 days). Naive animals were immunized with PBS and 20% Adjuplex™.

Blood was collected for evaluation of anti-cocaine antibody titers at indicated time points, allowed to clot, and centrifuged at 2500 *g* for 15 min. The resulting serum was stored at −80°C. Anti-cocaine antibody titers were measured by ELISA as described above and previously [11]. Blood was collected on cocaine administration days 178, 179, and 180 for evaluation of titer levels before and after cocaine administration.

For assessment of cocaine biodistribution, the nonhuman primates were anesthetized with ketamine (5 mg/kg) and dexmedetomidine (0.015 mg/kg) and challenged with 1 mg/kg cocaine delivered intravenously on days 178, 179, and 180 post primary vaccination. Animals were sacrificed 30 min following cocaine delivery on day 180. Euthanasia was accomplished by overdose of euthanasia solution, Euthasol (Virbac Animal Health, Fort Worth, TX), intravenously at 1 ml/4.5 kg (390 mg/ml Pentobarbital Sodium and 50 mg/ml Phenytoin Sodium) diluted in 1 ml saline, given by the attending veterinarian, consistent with the recommendations of the Panel on Euthanasia of the American Veterinary Medical Association. Importantly, no humane endpoints were necessary in this study. Potassium oxalate and sodium fluoride-treated serum was collected directly proceeding sacrifice and was stored at −20°C until the time of analysis. Following sacrifice, animals were perfused with 6 liters phosphate buffered saline, pH 7.4 and tissues were collected for storage at −80°C until the time of analysis. The level of cocaine and cocaine metabolites (benzoylecgonine, ecgonine methyl ester, and norcocaine) in the serum, brain (putamen), and heart were measured at The Center for Human Toxicology at the University of Utah, by liquid chromatography-electrospray ionization-tandem mass spectrometry (LC-ESI-MS/MS) as described previously [10].

### Statistical analysis

Sample sizes for the mouse biodistribution, mouse behavior, and nonhuman primate studies were determined by power analysis, based on data from De et al [12] and Hicks et al [10, 13], respectively. For nonhuman primate studies, animals received 50 to 200 μg of dAd5GNE and 20% Adjuplex. There were no significant differences in the titers or biodistribution of cocaine in animals receiving 50 μg of Ad5GNE and animals receiving 200 μg of dAd5GNE. For assessment of antibody titers and biodistribution, dose groups were combined. The specific parametric and non-parametric tests used for each study are summarized in the corresponding figure legends.

## Results

### dAd5GNE vaccination reduces cocaine levels in the brain following daily cocaine use

**Fig 1:**
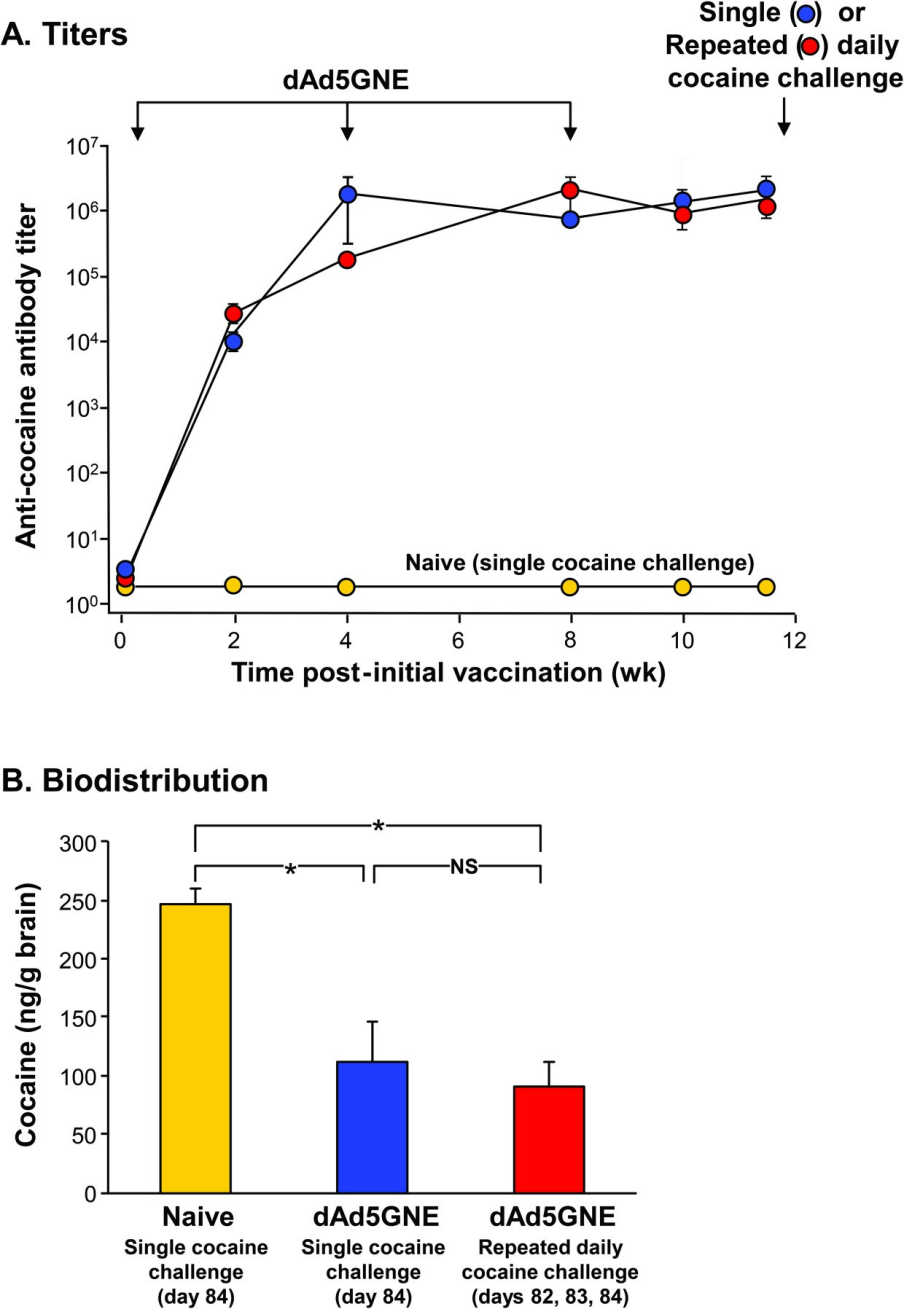
Vaccination with dAd5GNE evokes high titer antibodies and limits cocaine access to the brain following repeated daily cocaine use. **A.** Anti-cocaine antibody titer in dAd5GNE vaccinated mice. Naive animals had no detectable anti-cocaine antibody titer. **B.** Cocaine biodistribution following challenge with cocaine. Naive animals challenged with cocaine had high cocaine levels in the brain 1 min following a single 0.1 mg/kg cocaine challenge. dA5GNE vaccination significantly reduced cocaine levels in the brain 1 min following a single cocaine challenge. dAd5GNE vaccination also significantly reduced cocaine levels in the brain 1 min following the third of 3 repeated daily cocaine challenges. Results are shown as mean ± SE. Naive animals challenged with a single dose of cocaine are shown in yellow (n = 3). dAd5GNE vaccinated animals challenged with a single dose of cocaine are shown in blue (n = 4). dAd5GNE vaccinated animals challenged with 3 repeated daily cocaine challenges are shown in red (n = 5). Groups are compared by two-tailed unpaired t-tests (* denotes p<0.05, NS = not significant).

The cocaine vaccine dAd5GNE produced a high titer of anti-cocaine antibodies in mice (Fig 1A). Cocaine levels in the brains of dAd5GNE vaccinated mice receiving a single 0.1 mg/kg dose of ^3^H-cocaine were reduced by 55% when compared with cocaine levels in the brains of naive mice receiving a single 0.1 mg/kg dose of ^3^H-cocaine (p<0.05). Importantly, immunity induced by the vaccination continued to restrict cocaine from reaching the brain after 3 sequential daily doses of cocaine. After the third of 3 sequential daily doses of cocaine, the vaccinated animals had 64% less ^3^H-cocaine in the brain than naive animals after a single cocaine dose (Fig 1B). Notably, ^3^H-cocaine levels were similar in the brains of vaccinated mice receiving a single dose of cocaine and vaccinated mice receiving cocaine on 3 sequential days (p>0.5; Fig 1B).

### dAd5GNE reduces cocaine-induced hyperactivity and toxicity following repeated high-dose cocaine use

**Fig 2:**
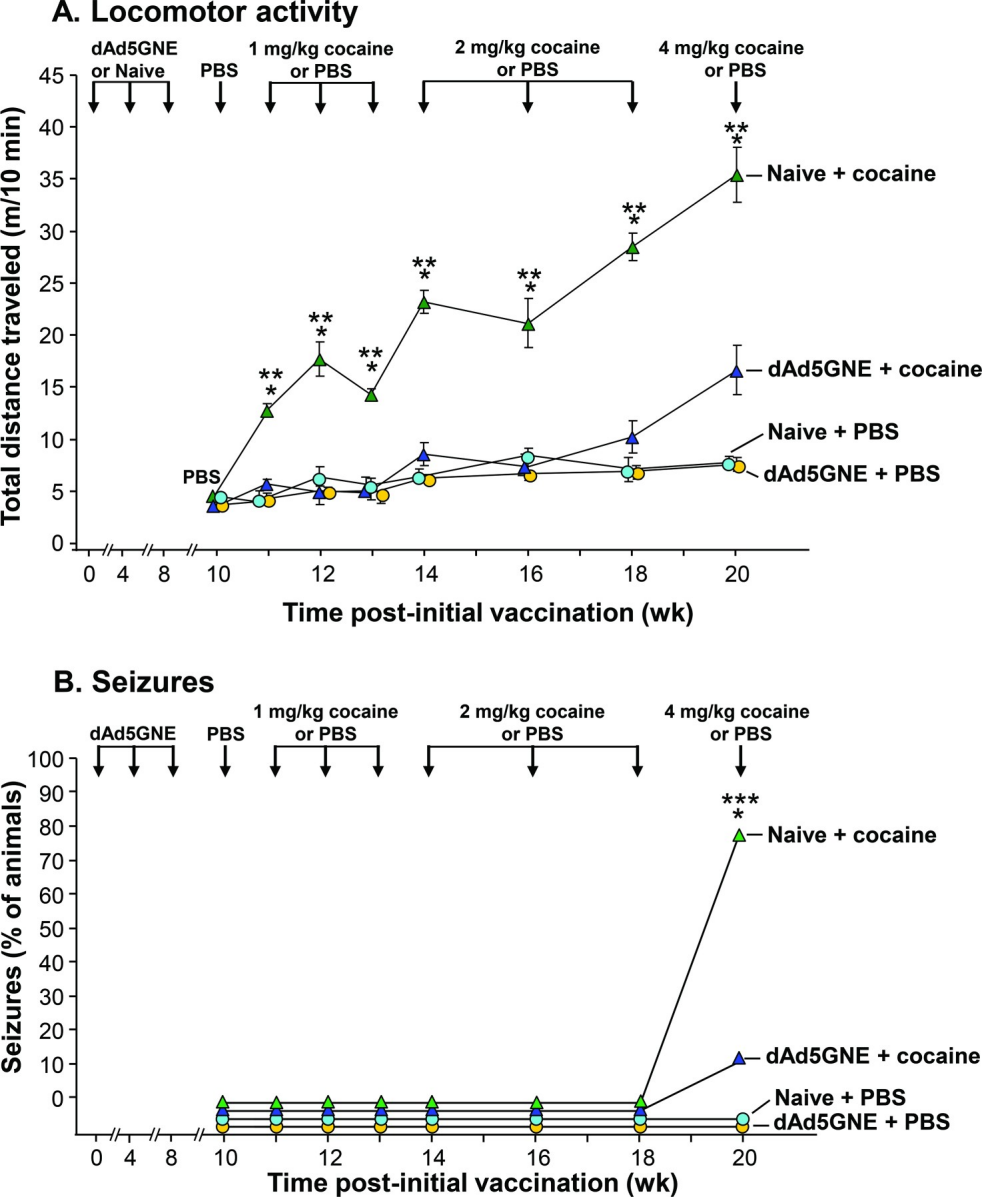
Vaccination with dAd5GNE reduces cocaine-induced hyperactivity and toxicity following repeated high-dose cocaine use. **A.** Total distance traveled. Mice were challenged with PBS 10 wk following initial vaccine administration, and then with PBS or cocaine (1 mg/kg on wk 11, 12, and 13; 2 mg/kg on wk 14, 16, and 18, 4 mg/kg on wk 20). The locomotor activity of naive and dAd5GNE vaccinated mice following each challenge is shown as mean total distance traveled in 10 min ± SE. Groups are compared by two-tailed unpaired t-tests. **B.** Seizure activity. The percentage of naive and dAd5GNE vaccinated mice experiencing a seizure following each challenge is shown as the percentage of animals experiencing a seizure within 10 min of administration. Groups are compared by one-tailed Barnard’s test. Naive + cocaine (green triangle; n = 5), dAd5GNE + cocaine (blue triangle; n = 10), naive + PBS (teal circle; n = 5), dAd5GNE + PBS (yellow circle; n = 10). * denotes p<0.05 for naive + cocaine vs naive + PBS. ** denotes p<0.05 for naive + cocaine vs dAd5GNE + cocaine in panel A. *** denotes p<0.01 for naive + cocaine *vs* dAd5GNE + cocaine in panel B. There were no significant differences between the dAd5GNE + cocaine and naive + PBS groups at any timepoint. There were no significant differences between the dAd5GNE + cocaine and dAd5GNE + PBS groups at any timepoint.

Cocaine administration will evoke both hyperlocomotor activity in mice and with escalating doses induce seizures [25]. We evaluated the capacity of the dAd5GNE vaccine to abrogate each. dAd5GNE vaccination did not influence the locomotor activity of mice following PBS mock drug challenge (p>0.2 naive + PBS *vs* dAd5GNE + PBS). However, the locomotor activity of naive mice was elevated following each of 1 mg/kg (p<0.05 *vs* naive + PBS), 2 mg/kg (p<0.005 *vs* naive + PBS), and 4 mg/kg (p<0.005 *vs* naive + PBS) cocaine administration. Conversely, the locomotor activity of dAd5GNE vaccinated mice was unchanged by all cocaine doses: 1 mg/kg (p>0.4 *vs* naive + PBS, p>0.2 vs dAd5GNE + PBS), 2 mg/kg (p>0.3 *vs* naive + PBS, p>0.2 *vs* dAd5GNE + PBS), and 4 mg/kg (p = 0.17 *vs* naive + PBS, p = 0.06 *vs* dAd5GNE + PBS). As such, vaccinated animals receiving cocaine were significantly less active than their naive counterparts at all cocaine doses: 1 mg/kg (p<0.01), 2 mg/kg (p<0.001), 4 mg/kg (p<0.05). Groups were compared by two-tailed unpaired t-tests, with each time-point compared independently (Fig 2A).

Notably, 80% of naive mice experienced epileptic seizures in response to the 4 mg/kg cocaine dose; only 10% of vaccinated animals experienced seizures when challenged with the same dose (p<0.01 naive + cocaine *vs* dAd5GNE + cocaine; Barnard’s test; Fig 2B). Animals challenged with PBS did not experience seizures (p = 0.39 naive + PBS *vs* dAd5GNE + cocaine; p = 0.26 dAd5GNE + PBS *vs* dAd5GNE + cocaine; Barnard’s test; Fig 2B).

### dAd5GNE induces anti-cocaine antibodies that remain high and reduce cocaine levels in the brain following daily cocaine use

**Fig 3:**
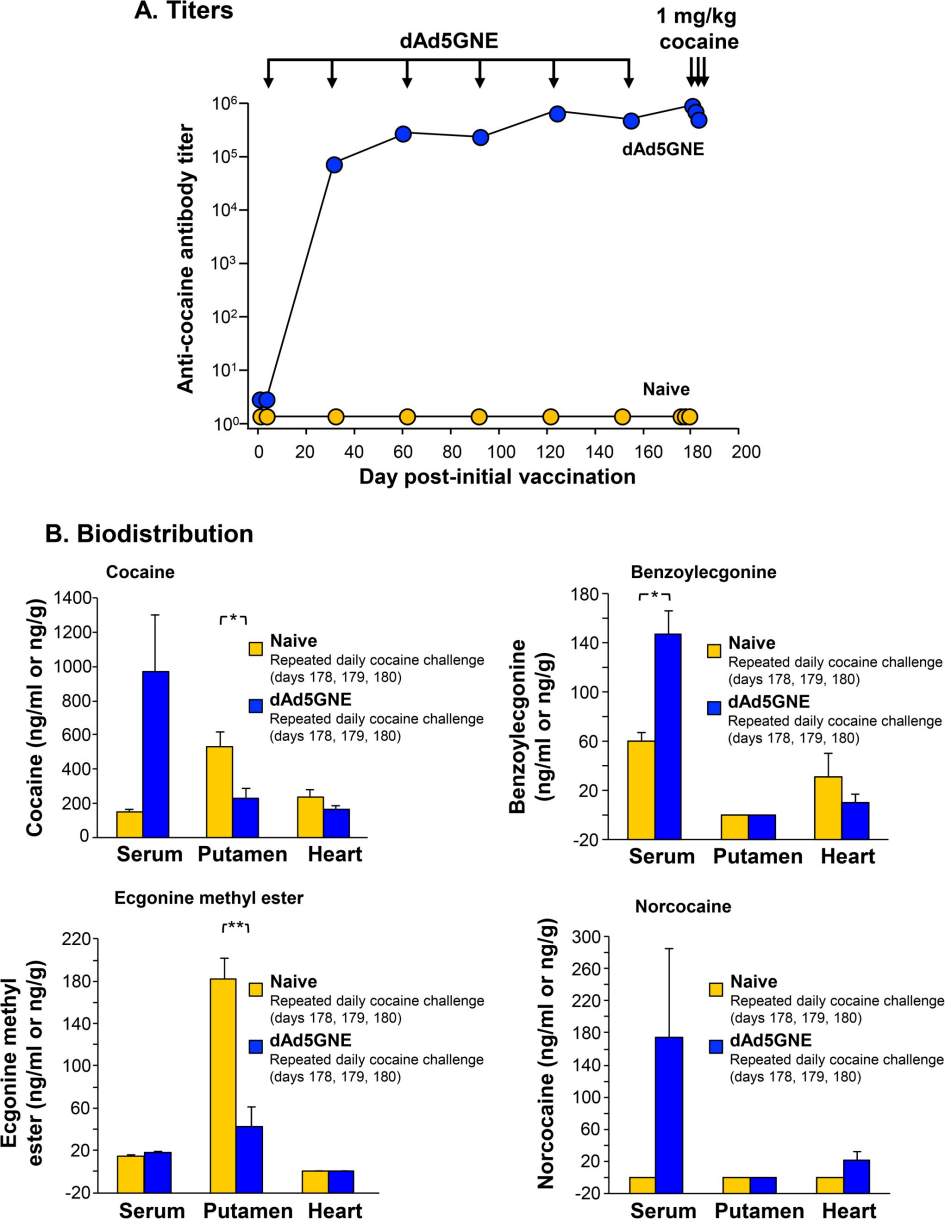
Daily cocaine administration to nonhuman primates. Vaccination with dAd5GNE induces anti-cocaine antibody titer that remains high and limits cocaine access to the brain following repeated daily cocaine administrations to nonhuman primates. **A.** dAd5GNE vaccinated non-human primates (n = 6M/6F) produced a high titer of anti-cocaine antibodies. Naive animals (n = 2M/2F) did not produce detectable anti-cocaine antibody titer. In dAd5GNE vaccinated animals anti-cocaine antibody titers remained high and were not significantly reduced following cocaine administration on day 178, 179, and 180. Groups are compared by two-tailed paired t-tests. **B.** Levels of cocaine and the cocaine metabolites benzoylecgonine, ecgonine methyl ester, and norcocaine in the serum, putamen, and heart were evaluated on day 180, 30 min following the last of three daily 1 mg/kg cocaine administrations on day 178, 179, and 180. All data is shown as mean concentration in ng/g or ng/ml ± SE. Groups are compared by two-tailed unpaired t-tests. (* p<0.05, ** p<0.01).

The cocaine vaccine dAd5GNE produced a high titer of anti-cocaine antibodies in nonhuman primates (Fig 3A). These titers remained high and were not significantly reduced by cocaine administration (Fig 3A). dAd5GNE vaccination significantly altered the biodistribution of cocaine in nonhuman primates following daily cocaine administration (Fig 3B). Cocaine levels in the putamen (brain) of vaccinated animals were reduced by 57% 30 min following the third of 3 daily 1 mg/kg cocaine challenges (p<0.05). There were trends toward tissue protection in the heart and increased cocaine levels in the serum of vaccinated animals. Similar results were seen with the cocaine metabolites benzoylecgonine and ecgonine methyl ester. Norcocaine levels trended higher in the serum and heart of vaccinated animals, but these results were not significant.

## Discussion

Cocaine produces a euphoric “high” by blocking the dopamine transporter (DAT) in the central nervous system (CNS), leading to the prolonged and intensified activation of neurons, mediated by dopamine accumulation in the synaptic cleft [19, 26–30]. The subjective rewards of a cocaine-induced “high” often trigger repeated use and frequently lead to drug dependency and addiction [19, 31]. While current therapies for cocaine addiction are limited to psychosocial interventions [32], the development of a pharmacological treatment that prevents the reinforcing effects of a cocaine-induced “high” holds promise as a potential clinical therapeutic for addicted individuals [3–5].

The cocaine vaccine dAd5GNE generates high titers of anti-cocaine antibodies, which block cocaine from reaching the CNS, and reduce the reinforcing effects of the drug [7, 10–13]. Our previous studies have demonstrated efficacy of this vaccine in the context of moderate and intermittent cocaine use [7, 10–13]. However, for the vaccine to be an effective therapy in clinical populations, the vaccine must be effective in the context of the repeated daily use and high-dose cocaine “binges” common for cocaine addicts [15–18, 20, 21].

While dAd5GNE has been shown to generate high titers of high-affinity anti-cocaine antibodies, multiple cocaine administrations may overwhelm the anti-cocaine immune response, rendering an individual vulnerable to successive cocaine administrations [33]. However, our data demonstrates that anti-cocaine antibody titers remain high following multiple moderate 1 mg/kg intravenous doses of cocaine. Anti-cocaine antibody titers trended downward after cocaine administration, but were not significantly different before and after cocaine administration, suggesting that individuals should still be protected before and after cocaine administration. These results are consistent with previous findings, which showed that the anti-cocaine antibody titers produced by cocaine vaccines are not significantly affected by cocaine exposure [34].

While the mechanism of vaccine resilience is currently under investigation, the data suggests that vaccination evokes anti-cocaine antibodies that are in pronounced molar excess to cocaine in the serum or that anti-cocaine antibodies are recycled and capable of protecting against multiple cocaine administrations [33]. In the models of daily use, it is possible that metabolism of cocaine reduces the burden for anti-cocaine antibodies to be available to protect against next day's dose. In this case, more frequent cocaine use could potentially overwhelm the immune response.

Although anti-cocaine antibody titers remain high following cocaine administration, for the therapy to be effective, the remaining antibodies must restrict multiple cocaine challenges from reaching the central nervous system. While previous studies have demonstrated that vaccination can reduce cocaine levels in the brain following intravenous administration, these studies have not directly compared single-dose exposures with multiple cocaine exposures and have not evaluated dAd5GNE as a potential therapy for daily cocaine users. Our data demonstrates that dAd5GNE protects the brain of vaccinated mice equally well in models of both repeated daily and single-dose cocaine use. This suggests that the cocaine vaccine dAd5GNE may be a lasting therapeutic for the substantial number of cocaine users who use the drug repeatedly on a daily basis. However, due to the range of assay sensitivity to the quantity of cocaine challenge, murine biodistribution studies only evaluated efficacy in the context of repeated daily low-dose cocaine use. To evaluate efficacy in the context of moderate and high-dose cocaine use, behavior studies were conducted in mice.

While reduction of cocaine in the brain is indicative of vaccine efficacy, an effective therapy must reduce DAT occupancy below 47% to prevent the reinforcing effects of a drug-induced “high” [19]. Our studies found that naive mice challenged with a moderate cocaine dose (1 mg/kg) demonstrated a significant increase in locomotor activity, suggesting that these animals were experiencing a cocaine-induced “high”. In contrast, vaccinated mice did not exhibit hyperactivity, suggesting that cocaine levels in the brain were reduced sufficiently to prevent a “high”.

However, one potential challenge to the efficacy of a cocaine vaccine is that a vaccinated individual may attempt to increase the dose of their cocaine use to overwhelm the anti-cocaine immune response [20, 21]. As such, success in addicted clinical populations may require the vaccine to abrogate atypically large doses of cocaine aimed at overriding the immune response. Our data indicates that increased doses of cocaine produced increased hyperactivity in naive animals, but did not significantly increase the activity of vaccinated animals, suggesting that dAd5GNE vaccination may protect individuals from the reinforcing effects of high-dose “binge” use.

In addition to the risk of continued addiction, high-dose cocaine use is associated with several dangerous side-effects such as drug-induced seizure and death [35–39]. We found that when intravenous doses of cocaine were increased to 4 mg/kg, 80% of naive mice experienced a cocaine-induced seizure. Interestingly, when vaccinated animals were challenged intravenously with the same cocaine dose, only 10% of vaccinated animals experienced a seizure, suggesting that vaccination not only prevents the stimulating effects of cocaine use, but also reduces the toxicity of high-dose use. Multiple 4 mg/kg doses of cocaine and doses greater than 4 mg/kg were not evaluated because a single administration of 4 mg/kg cocaine was sufficient to cause cocaine-induced seizures. However, protection from a single 4 mg/kg cocaine dose suggests that the vaccine would likely protect against doses that add to 4 mg/kg cocaine administered 20–30 min apart. This type of repeated drug use, at increasingly higher doses, within a relatively short period of time, would reflect a common multi-dose cocaine “binge” in humans.

While vaccinated mice challenged with 4 mg/kg cocaine demonstrated no significant increase in locomotor activity and were significantly protected from cocaine-induced seizures, at even higher doses the vaccine may be overwhelmed. Therefore, while high-dose cocaine use equivalent to human overdose may be significantly safer in a vaccination setting, vaccinees must be informed of the potential consequences of overwhelming the induced immunity. Additionally, while overwhelming the vaccine may be possible, previous studies have shown that when the cost of cocaine administration was increased dAd5GNE vaccinated animals exhibited decreased motivation to self-administer cocaine compared with control animals [7]. In humans, it is conceivable that the additional financial and social costs of high-dose cocaine use may shift the cost/benefit and risk/reward scales in favor of abstinence

Our data strongly suggests that dAd5GNE vaccination protects mice from repetitive and high-dose cocaine “binges.” The ability of these results to predict efficacy in humans is inherently limited by the comparability of small-animal models [40–42]. Notably, when compared to studies in mice, studies in nonhuman primates are considered more relevant to clinical translation of a cocaine vaccine [19, 43–47]. As such, the evaluation of vaccine efficacy in nonhuman primates presents an opportunity to substantiate findings established in mice. Mirroring the results in mice, cocaine levels in the brain (putamen) of vaccinated nonhuman primates were significantly reduced following repeated daily dosing with cocaine, consistent with the concept that dAd5GNE vaccination can be an effective therapy for daily cocaine users.

While cocaine levels in the brain of nonhuman primates were reduced following repeated moderate (1 mg/kg) daily cocaine challenges, one potential consequence of CNS protection is the accumulation of cocaine in visceral organs [48–53]. One organ of particular concern is the heart, where cocaine can interfere with action potentials, leading to arrhythmia and cardiovascular collapse [22, 54–58]. While this may be of particular concern in a vaccination setting, where cocaine is maintained systemically, our data indicates that cocaine levels in the heart are unaffected by vaccination (Fig 3B), suggesting that vaccination will not cause cardiovascular toxicities, even if addicts use the drug daily and at moderately high levels. Moreover, in our prior NHP studies, the vaccine protected the heart from administered cocaine with reduced levels compared to control animals, suggesting that the vaccine is protective of cardiovascular toxicities common in cocaine addicts [10]. The multiple administrations of the dAd5GNE vaccine in this NHP study was used to assure continuous evoked high titers so that the vaccine concept could be evaluated. Developing the minimum requirements for an efficacious human vaccination regimen is left to the clinical development pathway.

In summary, we conclude that dAd5GNE vaccination produces high titers of anti-cocaine antibodies, which persist following cocaine challenges, continues to protect the CNS from repeated daily cocaine use, and inhibits the cocaine-induced toxicities and the perceived “highs” stimulated by high-dose “binge” use. Based on these conclusions we hypothesize that if dAd5GNE produces equivalent high anti-cocaine antibody titers in human patients, the vaccine is likely to remain effective in the context of moderate daily cocaine use and high-dose cocaine “binges.” Based on these and other studies the FDA has allowed evaluation of dAd5GNE + 20% Adjuplex in a Phase I clinical trial (NCT02455479) [59].
